# Vacancy cluster in ZnO films grown by pulsed laser deposition

**DOI:** 10.1038/s41598-019-40029-3

**Published:** 2019-03-05

**Authors:** Zilan Wang, Caiqin Luo, W. Anwand, A. Wagner, M. Butterling, M. Azizar Rahman, Matthew R. Phillips, Cuong Ton-That, M. Younas, Shichen Su, Francis Chi-Chung Ling

**Affiliations:** 1 Department of Physics, The University of Hong Kong, Pokfulam Road, P. R. China; 2 Institute of Radiation Physics, Helmholtz-Zentrum Dresden-Rossendorf, Bautzner Landstr., 400, 01328 Dresden, Germany; 3 School of Mathematical and Physical Sciences, University of Technology Sydney, Ultimo, NSW 2007 Australia; 4 PGG, Physics Division, PINSTECH, P.O. Nilore, Islamabad, Pakistan; 5 Institute of Optoelectronic Material and Technology, South China Normal University, Guangzhou, 510631 P. R. China

## Abstract

Undoped and Ga-doped ZnO films were grown on c-sapphire using pulsed laser deposition (PLD) at the substrate temperature of 600 °C. Positron annihilation spectroscopy study (PAS) shows that the dominant V_Zn_-related defect in the as-grown undoped ZnO grown with relative low oxygen pressure P(O_2_) is a vacancy cluster (most likely a V_Zn_-nV_O_ complex with n = 2, 3) rather than the isolated V_Zn_ which has a lower formation energy. Annealing these samples at 900 °C induces out-diffusion of Zn from the ZnO film into the sapphire creating the V_Zn_ at the film/sapphire interface, which favors the formation of vacancy cluster containing relatively more V_Zn_. Increasing the P(O_2_) during growth also lead to the formation of the vacancy cluster with relatively more V_Zn_. For Ga-doped ZnO films, the oxygen pressure during growth has significant influence on the electron concentration and the microstructure of the V_Zn_-related defect. Green luminescence (GL) and yellow luminescence (YL) were identified in the cathodoluminescence study (CL) study, and both emission bands were quenched after hydrogen plasma treatment. The origin of the GL is discussed.

## Introduction

Zinc oxide is a wide band gap semiconductor having a direct bandgap of 3.33 eV that finds a variety of potential applications involving ultra-violet (UV) optoelectronic, spintronic, sensor and photovoltaic technologies^1^. ZnO has a high exciton density due to its large binding energy (~60 meV). Consequently ZnO is considered to be an excellent optoelectronic material for fabricating light emitting diodes and laser diodes operating in the UV spectral wavelength range. However, the development of ZnO-based optoelectronic devices is hindered by the asymmetric doping difficulty in ZnO, where the growth of n-type is straightforward while p-type remains a considerable challenge^2,3^. Recent studies suggest that p-type conductivity could be achieved in ZnO using intrinsic point defect complexes as shallow acceptors rather than the usual atomic substitution by dopants^4,5^. Implementation of this approach requires the development of fabrication procedures that provide precise control of the growth environment^5^ and a detailed knowledge of native point defects in ZnO. However, the nature of intrinsic point and complex defects in ZnO is poorly understood and remains controversial to date. For example, most first principal calculations report that V_O_ is a deep negative-U donor, but there is disagreement on whether V_O_ is present in n-type ZnO (ref. ^2^ and references therein). In undoped ZnO, V_Zn_ is shown to be a dominant compensating acceptor in the as-grown material^6^. Consequently, the incorporation of V_Zn_, V_O_ and their defect complexes should significantly change the electrical, optical and magnetic properties of ZnO. Conversely, ZnO films doped with group III metals like Ga and Al can have low electrical resistivity and high optical transmittance, thus are excellent candidates for the transparent electrode applications in photovoltaics, optoelectronics and transparent electronics. Recent studies show that the degenerate doping of Ga in ZnO induces the formation of a high concentration of V_Zn_-related defect acceptor due to lower V_Zn_ formation energy, which leads to the self-compensation of the n-type doping^7^. There are also theoretical and experimental reports on the formation of shallow acceptor complex (As_Zn_V_Zn_) in As-doped ZnO and similar complexes in P and Sb doped ZnO^8,9^. Additionally, the V_Zn_ and V_O_ has been suggested to be associated with the room temperature ferromagnetism observed in undoped ZnO and transition metal doped ZnO^10,11^. However, there are relatively few reports on the study of vacancy clusters in ZnO^12–15^. Significantly the majority of these studies identified vacancy cluster induced during ion-implantation and the subsequent annealing in the ZnO samples, rather than in both as-grown and annealed non-irradiated samples.

Positron annihilation spectroscopy (PAS) is a probe for atomic-scale open volume defects in semiconductors^16,17^. Positrons incident into the solid are rapidly thermalized and then undergo random walk in the delocalized bulk state. Positron would be trapped into a neutral or negatively charged open volume defect as the missing lattice atomic core is a potential well as seen by the positively charge positron. The Doppler broadening of the outgoing gamma photons and the positron lifetime reveal the electronic momentum distribution and the electronic density which are the fingerprints of the open volume defects where the positrons annihilate. PAS is thus selectively sensitive to the presence of vacancy-type defect and capable of revealing the defect’s microstructure (defect configuration). Although PAS has been extensively utilized to investigate and understand Zn-vacancy related defects in ZnO single crystal (for examples references^6,18–22^), there have been relatively only a few PAS studies on ZnO films grown on substrates^23–26^, which is indeed the active layer for many device applications. In our previous work, defects containing V_Zn_ were identified in PLD grown undoped ZnO samples grown at the sapphire substrate temperature of 300 °C^23^. However, the identities and defect configurations and their interactions during annealing were not determined.

In the present study, V_Zn_-related defects in the as-grown ZnO films grown on c-plane sapphire by Pulsed Laser Deposition (PLD) with a substrate temperature of 600 °C were investigated by Doppler broadening spectroscopy (DBS). The electrical and optical properties of the films were studied by Hall effect measurement and cathodoluminescence (CL). The film-substrate interface was studied by the high resolution transmission electron microscopy (HRTEM). The combination of these techniques reveals that a vacancy cluster is the dominant positron trapping defect in the PLD grown ZnO films and the microstructure of this vacancy cluster depends on the oxygen content during growth, post-growth annealing and degenerate Ga-doping. The effect of the vacancy cluster on the electrical and optical properties of the films are discussed.

### Experimental

The ZnO film samples were grown using PLD on a c-plane sapphire substrate with a chamber background pressure of 10^−4^ Pa. The pulse energy of the 248 nm laser pulse from the Coherent COMPexPro 102 excimer laser was 300 mJ. The substrate temperature was 600 °C during the growth. In order to study for the effect of the O-abundance during the growth, undoped samples were grown using a pure ZnO ceramic target with the oxygen pressure P(O_2_) of 0 Pa, 0.02 Pa, 1.3 Pa and 5 Pa. These samples have moderate electron concentration ~10^18^ cm^−3^. In order to study the effect of Ga-doping and oxygen growth pressure, n^+^-samples namely the Ga-doped and Ga-Cu co-doped ZnO samples having n^+^~10^20^ cm^−3^ were fabricated by ZnO:Ga (Ga content = 1% weight ratio) and ZnO:Ga:Cu (Ga and Cu contents = 1% and 2% respectively in weight) with P(O_2_) = 0.02 Pa. Two control samples with moderate electron concentrations and higher P(O_2_) were also fabricated. One is the Cu-doped ZnO sample grown by the target with Cu content = 2% weight ratio at P(O_2_) = 0.02 Pa. The other control sample is the Ga-Cu co-doped ZnO sample grown with the same weight ratio of Ga and Cu as the degenerate Ga-Cu co-doped but with high P(O_2_) = 0.5 Pa and this yielded a Ga-Cu co-doped ZnO sample with moderate electron concentration. Isochronal annealing was carried out in a tube furnace for the time period of 40 minutes in Ar atmosphere over a temperature range of 700 °C to 900 °C. The XRD measurements were performed using a Siemens D5000 diffractometer with the CuKα line. Room temperature Hall effect measurements were made using the Accent HL-5500PC system. High resolution transmission electron microscopy (HRTEM) in a JEOL 2010F HRTEM was used to study the film/substrate interface of the sample.

The CL measurements were carried out at 80 K using the FEI Quanta 200 scanning electron microscope (SEM) equipped with a high resolution Hamatmatsu S7011-1077 CCD image sensor. For depth-resolved analysis, the electron beam energy was varied from 2 keV to 10 keV while the excitation power was kept constant at 30 μW.

Positron Doppler Broadening Spectroscopy (DBS) measurements were carried out using a continuous mono-energetic positron beam having a maximum energy of 35 keV. The annihilation gamma ray energy spectrum is collected by a high purity Ge detector system having an energy resolution of 1.09 keV for the 511 keV gamma line. The Doppler broadening of the gamma ray spectrum is monitored by the S-parameter and W-parameter defined respectively as the counts of the central window (511.00 ± 0.76 keV) and the wing windows (511.00 ± 3.4 keV and 511.00 ± 6.8 keV) to the count of the annihilation peak.

## Results and Discussions

**Figure 1:**
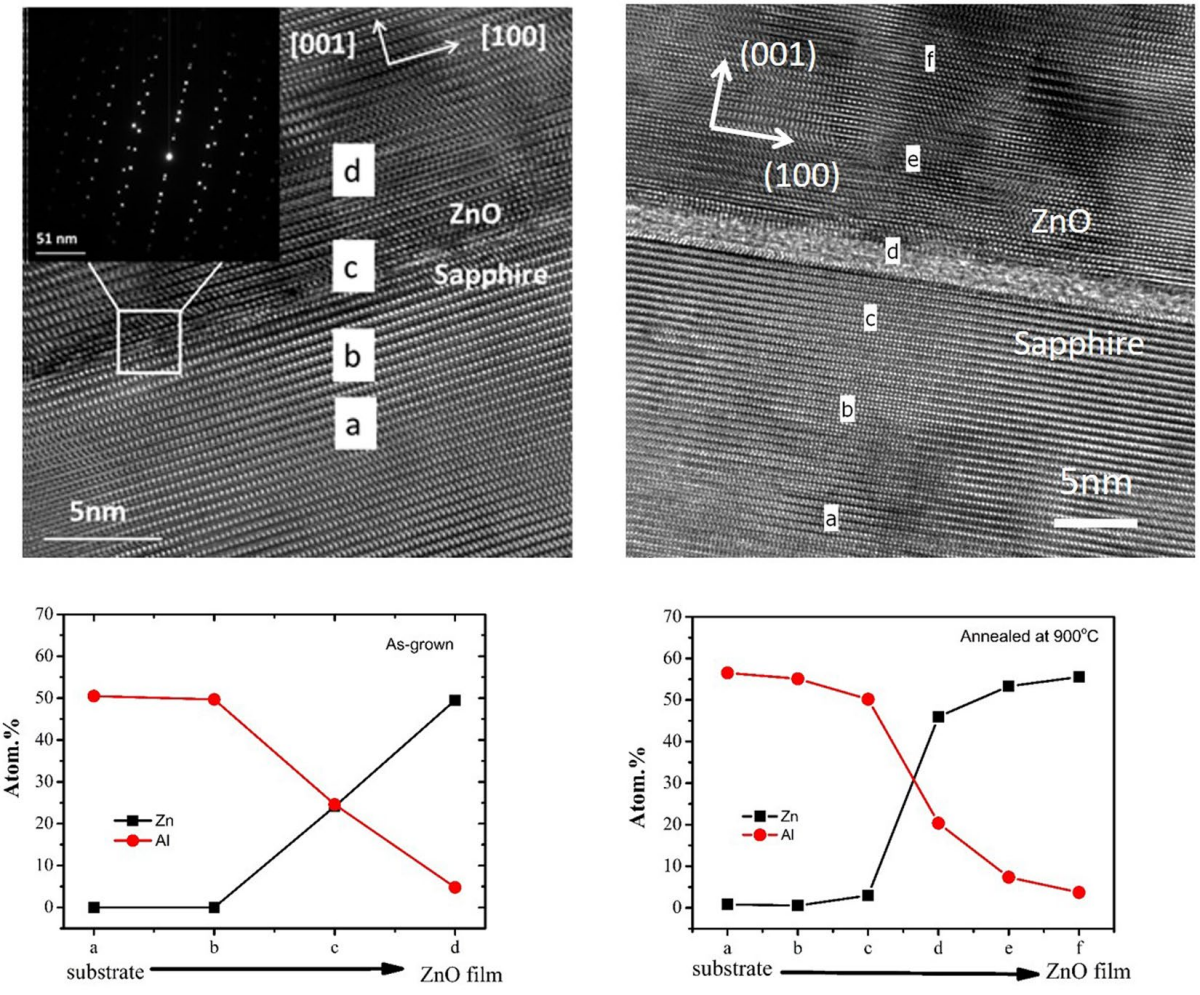
Cross-sectional HRTEM images of the ZnO-sapphire interface for the as-grown undoped ZnO grown with P(O2) = 0 Pa and that after annealing at 900 °C. The atomic profiles of Zn, O and Al obtained by EDXS taken at the different points on either side of the interface are also shown, indicating the inter-diffusion of Zn and Al across the film/substrate interface.

XRD spectra of all the ZnO film samples revealed only characteristic peaks for ZnO: (002) at 34.5°, sapphire (006) at 41.7° and ZnO (004) at 72.1°, indicating that the ZnO films had the single-phase wurtzite structure (XRD spectra shown in Fig. S1 of Supplemental Information). The HRTEM image and the atomic profiles of Zn and Al across the film/sapphire boundary obtained by Energy-Dispersive X-ray Spectroscopy (EDXS) for the as-grown undoped ZnO grown with P(O_2_) = 0.02 Pa and that after annealing at 900 °C are shown in Fig. 1. For the as-grown film, the HRTEM image shows a periodic ZnO lattice growth along the (002) plane. Both the HRTEM image as well as the EDXS profile indicate that the interface is atomically abrupt. After annealing at 900 °C, the interface becomes non-abrupt, indicating the inter-diffusion of Zn and Al across the interface.

The electron concentrations for the as-grown ZnO films (undoped, Cu-doped, Ga-doped and Cu-Ga co-doped) grown at different oxygen pressure are presented in Table 1. The undoped ZnO films grown with P(O_2_) = 0 to 5 Pa and the Cu-doped film (2 wt% in the deposition target) grown with P(O_2_) = 0.02 Pa have electron concentrations of 3–8 × 10^18^ cm^−3^ (±1 × 10^18^ cm^3^). For the Ga-doped (1 wt%) and Ga(1 wt%)-Cu(2 wt%) co-doped ZnO films grown at P(O_2_) = 0.02 Pa, the electron concentration is much higher, n^+^∼4–6 × 10^20^ cm^−3^ since Ga is a shallow donor in ZnO. However, growing the Ga(1 wt%)-Cu(2 wt%) co-doped ZnO samples at a higher P(O_2_) of 0.5 Pa yields the electron concentration of 8 × 10^18^ cm^−3^.

**Table 1:** The electron concentrations of the as-grown undoped ZnO, ZnO:Cu(2%), ZnO:Ga(1%) and ZnO:Ga(1%):Cu(2%) samples grown with different oxygen pressures.

	Oxygen Pressure (Pa)	n (cm^−3^)
ZnO	0	6 × 10^18^
	0.02	6 × 10^18^
	1	3 × 10^18^
	5	8 × 10^18^
Cu-doped ZnO (2%)	0.02	3 × 10^18^
Ga-doped ZnO(1%)	0.02	6 × 10^20^
Cu, Ga co-doped ZnO (Cu 2%, Ga 1%)	0.02	4 × 10^20^
Cu, Ga co-doped ZnO (Cu 2%, Ga 1%)	0.5	8 × 10^18^

**Figure 2:**
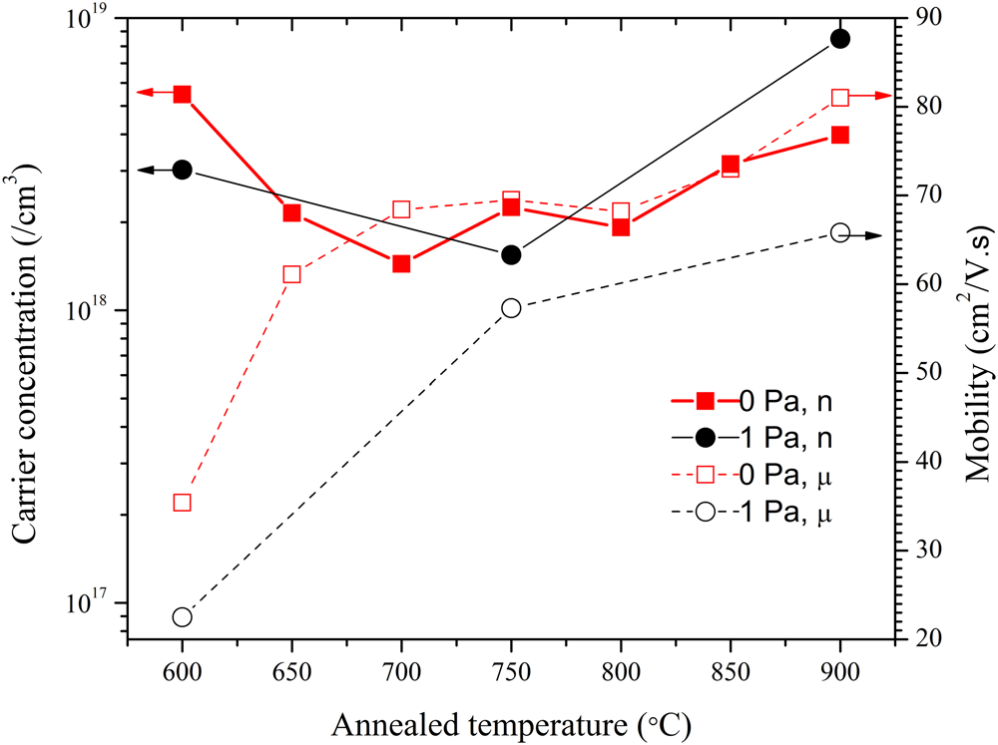
Carrier concentrations as a function of the annealing temperature for the undoped ZnO samples grown with P(O_2_) = 0 and 1 Pa.

Figure 2 shows the electron concentration (n) and mobility (μ) of the undoped ZnO samples grown with P(O_2_) = 0 and 1 Pa as a function of the annealing temperature. For the sample grown without oxygen, the electron concentration, n, is 6 × 10^18^ cm^−3^ for the as-grown sample, and it decreases to 2 × 10^18^ cm^−3^ after annealing at 650 °C. No significant change in the carrier concentration was observed with increasing annealing temperature up to 800 °C. However, further increase of the annealing temperature to 900 °C was found to increase the carrier concentration to ∼4 × 10^18^ cm^−3^. The electron mobility, μ, was observed to increase from ∼35 cmV^−2^ s^−1^ for the as-grown sample to ∼70 cmV^−2^ s^−1^ after annealing at 700 °C. No change in the mobility was found when the annealing temperature was increased to 800 °C, however, an increase to 81 cmV^−2^ s^−1^ occurred at 900 °C. Similar changes in n and μ with increasing annealing temperature were found in the undoped ZnO sample grown with P(O_2_) = 1 Pa.

**Figure 3:**
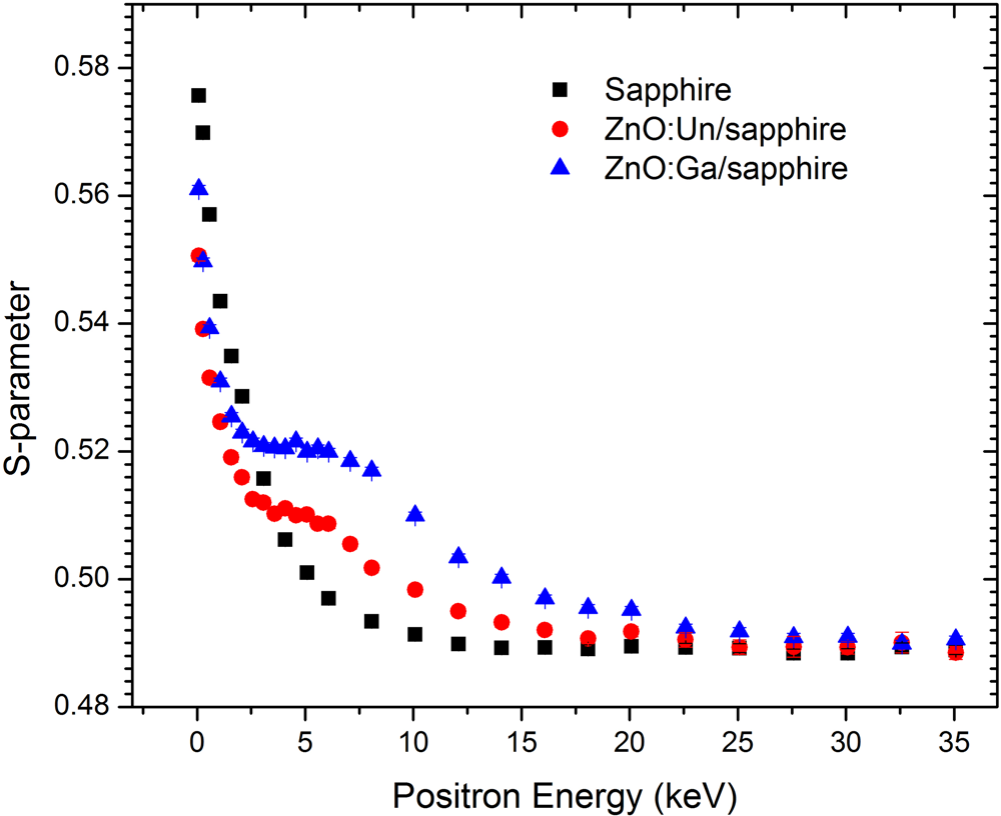
The S-parameter versus positron implantation energy (*S-E*) plots of the sapphire substrate, the undoped and Ga-doped (1 wt%) ZnO films grown on sapphire with P(O_2_) = 0.02 Pa.

**Figure 4:**
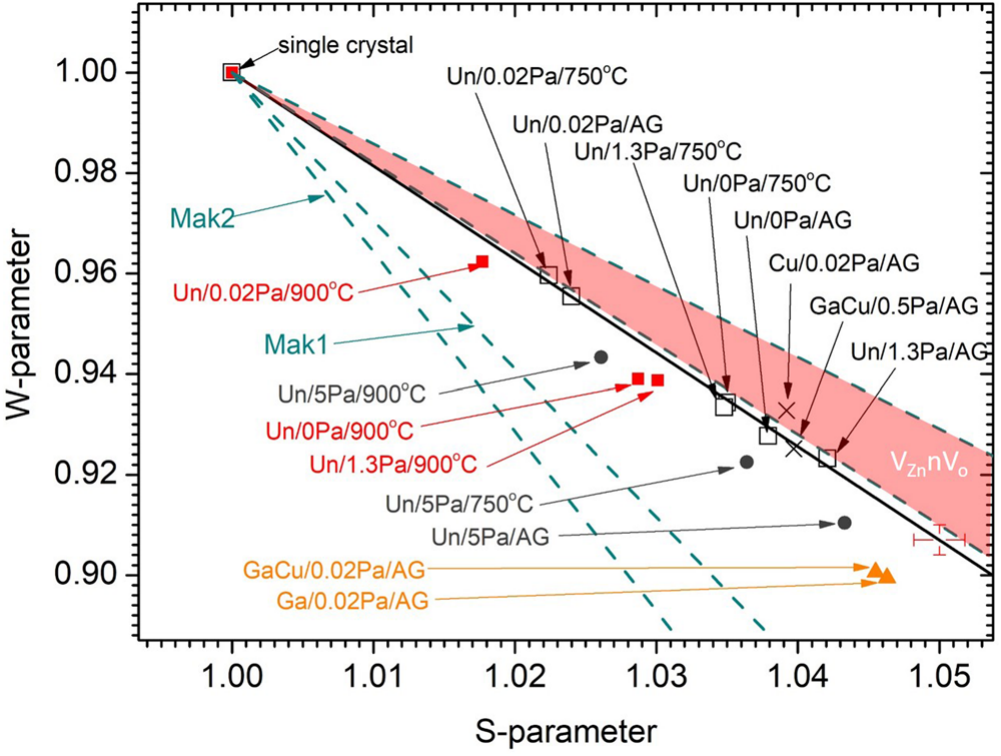
The *W-S* parameter plots of the different ZnO films and the error bars of the *W-S* data are shown at the low right hand corner. The *S* and *W*-parameters are normalized against those of the ZnO single crystal (data included in the figure), which is close to defect-free. The (*W,S*) data for the as-grown and 750 °C annealed undoped ZnO samples grown with relatively low P(O_2_) ≤ 1.3 Pa (dark open square) all lay on the straight line V1 (dark line) with excellent fitted *R*-square (0.99). The (*W,S*) data of the undoped ZnO samples grown with P(O_2_) ≤ 1.3 Pa and annealed at 900 °C are denoted by red solid square and are statistically distinguishable from V1 (argument given in text). The (*W,S*) data of the different Ga and Cu-doped ZnO samples are also included in the figure. Line Mak1 is the characteristic *W-S* line for V_Zn_, V_Zn_V_O_ and 2V_Zn_-V_O_ given in the theoretical study of Makkonen *et al*.^27^, for which they are not distinguishable by the DBS. Mak2 is the theoretical *W-S* line of (V_Zn_)_4._ The theoretical W-S lines for V_Zn_-nV_O_ (n = 2, 3) with different configurations are close and lay within the red region.

PAS is selectively sensitive to Zn-vacancy related defects in ZnO^16^. Depth profiling of S and W-parameters were conducted on the ZnO films fabricated with different P(O_2_), annealed with different temperatures and with different dopings. Typical *S-*parameter versus positron incident energy (*S*-*E*) plots for the undoped ZnO grown with P(O_2_) = 0.02 Pa, the Ga-doped (1%) ZnO grown with P(O_2_) = 0.02 Pa and the sapphire substrate, are shown in Fig. 3. The mean positron implanted depth is given by *x* = *AE*^1.6^/*ρ*, where *ρ* is the materials density and *A* = 4.0 μgcm^−2^ keV^−1.6 ^^15,16^. The *S-E* curves of the two ZnO/sapphire samples decreased with increasing *E* for small *E* < 2.5 keV. This is because as *E* increases, fewer positrons annihilate at the surface and more positrons annihilate in the ZnO film, and the *S-*parameter of the surface is larger than that of the film. For 2.5 keV < *E* < 7 keV, the *S-E* curves show a plateau indicating that all the implanted positrons annihilated in the film. A further increase of *E* decreases the *S*-parameter and finally the *S*-parameter saturates at *E* ≈2  keV, signifying that all positrons are now annihilated inside the sapphire. The average positron implanted depth at the positron incident energy *E* = 5 keV is ~140 nm. As the film thickness is ~200–300 nm, the leveling off at ~5 keV of the *S-E* data for the three samples as seen from Fig. 3 characterize the S-parameters of the annihilation gamma radiation predominately coming from the corresponding films. Consequently, the *S* and *W*-parameters obtained for each of the samples were normalized against those acquired from the ZnO single crystal and the *W*-parameters were plotted against the *S*-parameter as shown in Fig. 4. The positron lifetime spectrum of the bulk single crystal used for the normalization reference was well fitted by a single lifetime component of 166 ps, indicating that the sample is close to defect free^6,18–20^.

The positrons with energy of 5 keV implanted into the samples are rapidly thermalized inside the film, and undergo diffusion in the delocalized state. Subsequent trapping by a neutral or negatively charged open volume defect, if the defect exists, can then occur annihilating the positron with either a surrounding electron in the delocalized bulk state or the localized defect state. The measured *S*-parameter is given by $\,S={f}_{b}{S}_{b}+\sum _{i}{f}_{d,i}{S}_{d,i}$^16,17^, where *f*_*b*_ is the fraction of positron annihilating in the delocalized bulk state and *S*_*b*_ is its characteristic *S*-parameter. *S*_*d,i*_ and *f*_*d,i*_ are respectively the characteristic *S*-parameter and the fraction of positron annihilating in the *i*-th defect state. Similar relation also holds for the *W*-parameter, i.e.$\,W={f}_{b}{W}_{b}+\sum _{i}\,{f}_{d,i}{W}_{d,i}$. If a single type of defect exists, the equations can be combined, yielding $(S-{S}_{b})/(W-{W}_{b})=({S}_{d}-{S}_{b})/({W}_{d}-{W}_{b})$^16,17^. This equation implies that if there is single type of defect, the *W-S* plot will be a straight line passing through the point (*W*_*b*_,*S*_*b*_)and its slope is a characteristic parameter of the defect^16,17^.

Figure 4 shows the *W-S* data of the undoped ZnO samples grown with different oxygen pressures and subjected to different post-growth annealing treatments. It also contains the W-S data points of the ZnO single crystal, the as-grown Cu-doped(2%), Ga(1%)-doped and Ga(1%)-Cu(2%)-co-doped ZnO samples with moderate and degenerate electron concentrations. The experimental error of the *S* and *W*-parameter are indicated by the error bars at the lower right-hand corner of Fig. 4. This graph shows that the data points of the as grown and 750 °C-annealed undoped ZnO films grown with relatively low P(O_2_) = 0 Pa, 0.02 Pa and 1.3 Pa (black open square symbols in Fig. 4) as well as that of the ZnO single crystal lay on a straight line. Linear regression analysis of these data gave a fitted slope of −1.88 ±0.04 with an adjusted R squared value of 0.99. Accordingly, it is reasonable to conclude that samples belonging to this series result from the same type of V_Zn_-related defect, i.e. the V1 center. For samples grown with the same P(O_2_), annealing at 750 °C has the effect of moving the *W-S* data point up along the V1 straight line (i.e. decreasing the *S*-parameter and increasing the *W*-parameter), indicating a decrease in the V1 concentration without any change in the defect microstructure.

The *W-S* data points are observed to move below the V1 straight line (the solid red square symbols in Fig. 4) in these undoped samples grown with relatively low P(O_2_) when annealed up to 900 °C. The other series of samples, namely the undoped ZnO samples grown with the high P(O_2_) = 5 Pa with different annealing treatments (solid dark circles), and the n^+^ Ga-doped and Ga-Cu-doped ZnO samples (red solid triangles) also have their *W-S* data away and below the V1 straight line. As it is noticed that these data points though away from the V1 line but still only marginally separable while considering the experimental error, it is valuable to analyze the corresponding *W-S* line slopes while joining with the defect-free ZnO single crystal (*W,S*) data. The *W-S* data of these samples while joined with the single crystal W-S data have straight lines with slopes between −2.03 and −2.20, which is statistically different from that of V1 (−1.88 ±0.04). It is plausible to conclude that these samples contain V_Zn_-related defects other than V1.

Makkonen *et al*.^27^ carried out the theoretical study of Doppler broadening on V_Zn_ and its relevant clusters in ZnO. It is worthy to compare the present data and the theoretical characteristic *W-S* lines of the various vacancy clusters. A theoretical *W-S* straight line of V_Zn_ is plotted in Fig. 4 and labelled Mak1, using its characteristic Doppler broadening parameters (W,S)=(0.82,1.06), which was obtained from the W-S plot in ref. ^27^. It is also found that the theoretical (*W,S*) data points of the vacancy clusters V_Zn_-V_O_ and 2V_Zn_-V_O_ are closely positioned to those of V_Zn_. The (*W,S*) values of the V_Zn_-rich 4V_Zn_-V_O_ is ~ (0.75,1.1) which is also adjacent to the line Mak1. Therefore taking into account the resolution (see error bars in Fig. 4) of the Doppler broadening spectroscopy (DBS), isolated V_Zn_, V_Zn_V_O_ divacancy, the V_Zn_-rich 2V_Zn_-V_O_ and 4V_Zn_-V_O_ are not easily differentiated by this technique. The effect of adding V_O_ to the vacancy cluster would horizontally shift the (*W,S*) point to the right side (i.e. an increase of *S*-parameter), while adding a V_Zn_ moved the (*W,S*) point vertically down (i.e. decrease of *W*-parameter). The 4V_Zn_ vacancy cluster has (W,S)=(1.08,0.71) and its corresponding line is included as Mak2 in Fig. 4 and is clearly below Mak1, indicating that 4V_Zn_ and V_Zn_ centers can be distinguished using DBS. The region shaded in red in Fig. 4 is associated with the (S,W) of the V_O_-rich V_Zn_-nV_O_ defects with n = 2,3.

The (W,S) points of the as-grown undoped ZnO grown with relatively low P(O_2_) and those annealed at 750 °C in this work both lie on the straight line V1. It was reported that the experimental(W,S) points of the N-implanted ZnO samples and after annealing reported was very close to the theoretical W-S lines of V_Zn_-nV_O_ with n = 2,3 ^15,27^. As the present V1 line is very close to that of the vacancy cluster found in the ion-implanted as well as to the theoretical line of V_Zn_-nV_O_ (n = 2,3), it is plausible to suggest that the V1 is the V_Zn_-nV_O_ with n = 2,3.

Using the VEPFIT source code^28^, the effective positron diffusion lengths, L_+_, for these undoped ZnO samples were obtaining by fitting their *S-E* data with a two-layer model, giving respectively *L*_+_ = 12 to 23 nm (with errors of ~±1 nm) depending on P(O_2_) and annealing temperature. The fitted boundary position coincides well with the physical thicknesses of the films (200–300 nm depending on P(O_2_)). The defect concentrations, *C*_*V*_, can be estimated by the equation *C*_*v*_ = $[{({L}_{+,B}/{L}_{+})}^{2}-1]/\mu {\tau }_{B}$ ^12^, where *L*_+,*B*_ is the positron diffusion length of the defect free material, *τ*_*B*_, is the characteristic bulk lifetime and *μ* is the positron trapping coefficient. Although *μ* for V_Zn_-nV_O_ with n = 2,3 is not known precisely, an estimate of the order of magnitude of the V_Zn_-nV_O_ concentration can be obtained using a representative value of *μ*~10^16^ s^−1^ while *μ* is reported to be 3 × 10^15^ s^−1^ for V_Ga_ in GaN^29^. Using *τ*_*B*_ as 166 ps, which is value of the positron lifetime of the single component of the positron lifetime spectrum of our ZnO single crystal, and *L*_+*,B*_~70 nm (values obtained from the ZnO single crystal sample and references^18–22^), *C*_*V*_ for V_Zn_-nV_O_ found in these samples is ~3 ×10^17^ cm^−3^ for taking L_+_~18 nm.

V_Zn_ has also been identified in as-grown undoped ZnO single crystals^6^, ZnO films grown by atomic layer deposition (ALD), metalorganic chemical vapor deposition and molecular beam epitaxy (MBE)^7,24–26^. The vacancy cluster concentration as found in the as-grown PLD grown sample in this work is higher than those found in the single crystal (~10^15^ cm^−3^), but comparable to the films grown by ALD, MOCVD and MBE (~10^17^ cm^−3^).

For the undoped ZnO samples grown with relatively low P(O_2_) ≤ 1.3 Pa, annealing these samples at 900 °C have the effect of shifting the (*W,S*) points on the V1 line downward into the yellow shaded region. According to theoretical results^27^, this indicates the presence of vacancy clusters having more V_Zn_ as relative to the original V_Zn_-nV_O_ after the 900 °C annealing process. Annealing induces diffusion of the Zn vacancies. Once mobile, the Zn vacancy can move to a sink (for example the surface) and vanish or alternatively it can diffuse to another vacancy to form a vacancy cluster. Zn out-diffusion from the ZnO film to the Al_2_O_3_ side across a ZnO/Al_2_O_3_ interface during annealing has been observed experimentally^30^. Furthermore, our HRTEM and EDXS studies on the film/substrate interfaces of the undoped ZnO samples confirm the inter-diffusion at the interface after annealing at 900 °C (Fig. 1). The Zn out-diffusion from the ZnO film to the sapphire substrate would inevitably create V_Zn_ on the film side. Consequently the V_Zn_-rich environment would lead to the vacancy cluster with microstructure containing more V_Zn_ as relative to V1 thus pulling the (*W,S*) data point vertically down after annealing at 900 °C.

For the ZnO sample series grown with higher P(O_2_) = 5 Pa (as-grown and annealed, dark circles in Fig. 4), the (*W,S*) points are all below the V1 straight line and are also located in the yellow shaded region. High P(O_2_) during O-rich growth may lead to the formation of vacancy cluster with more V_Zn_ and less V_O_, thus pulling down the experimental (*W,S*) data point. It is worthy to point out that the observed (*W,S*) parameters in the yellow region do not coincide with any of the theoretical characteristic lines of vacancy clusters^27^. It may be the effect of co-existence of V1 and other V_Zn_-richer vacancy cluster, but of course we cannot exclude the possibility of formation of defects not studied in the theoretical study^30^.

For the Ga doped and Ga-Cu co-doped samples grown at relatively low P(O_2_) = 0.02 Pa having a degenerate electron concentration of n^+^~10^20^ cm^−3^, their (*W,S*) points also lie within the yellow shaded region. However for the Cu-doped sample grown with the same P(O_2_) and the Ga-Cu co-doped ZnO sample grown with the higher P(O_2_) = 0.5 Pa which have n ∼10^18^ cm^−3^, the (*W,S*) points are positioned on or above the V1 straight line. This implies that with the same Ga doping level, low P(O_2_) leads to degenerate electron concentration and the formation of a vacancy cluster having more V_Zn_ as compared to V1 (i.e. V_Zn_-nV_O,_ n = 2, 3). The higher tendency for forming a V_Zn_-rich vacancy cluster in degenerate n^+^ samples may be associated with the favorability of V_Zn_ in degenerate ZnO, for which enhancement of V_Zn_ concentration has been reported in Ga-doped degenerate ZnO samples^7^. However, we cannot exclude the possibility that the difference in the W-S data for the Ga-doped grown in different P(O_2_) is related to the formation of Ga_Zn_-V_Zn_, which as a deep acceptor is more energetically favorable than V_Zn_^31^. Electron paramagnetic resonance (EPR) studies have revealed that Al in ZnO binds with 90% of the electron irradiation induced V_Zn_ to form Al_Zn_-V_Zn_ in ZnO single crystal^31^. Higher P(O_2_) during growth may result in more V_Zn_ and thus more Ga_Zn_-V_Zn_ complexes, which seems to be consistent with the observed decrease of electron concentration from ~10^20^ cm^−3^ to ~10^18^ cm^−3^ in the Ga-codoped ZnO samples when the P(O_2_) increases from P(O_2_) = 0.02 Pa to 0.5 Pa. The observation of the (*W,S*) data shifting up to the V1 line could be expected if the (*W,S*) characteristic line of the Ga_Zn_-V_Zn_ is close to V1. Nevertheless, further investigation is needed to confirm this behaviour.

**Figure 5:**
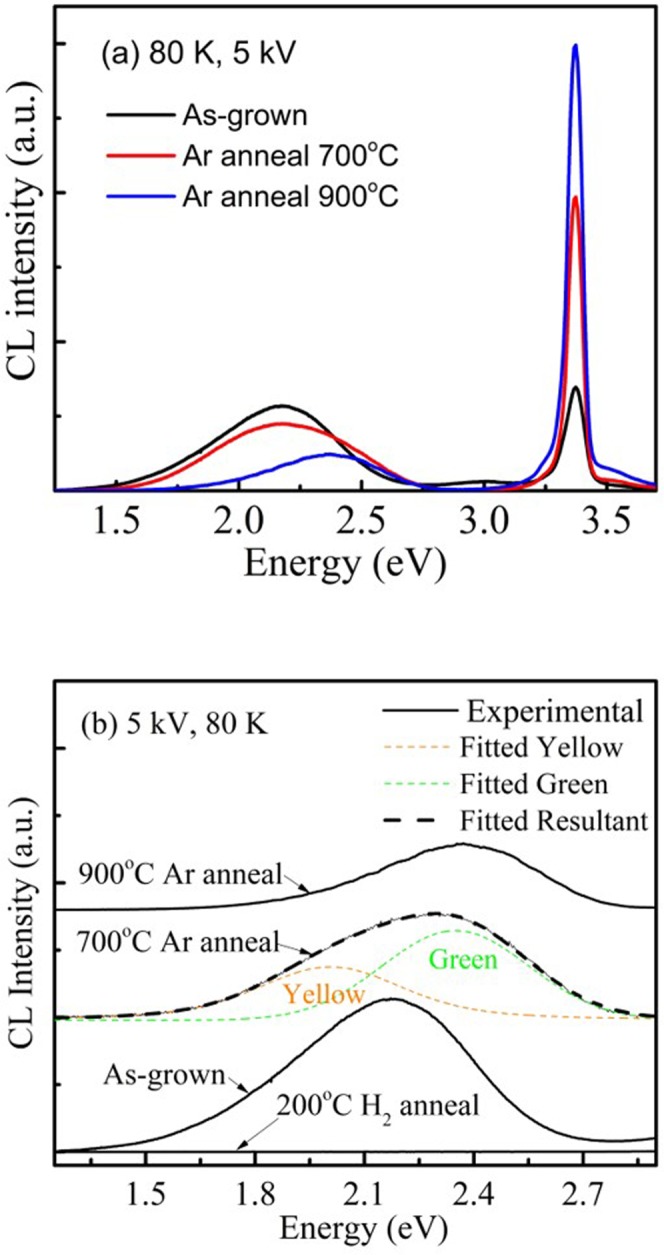
(**a**) The CL spectra of the as-grown, 700 °C and 900 °C annealed undoped ZnO samples grown with P(O_2_) = 0 Pa. (**b**) The modelled CL spectra fitted with the yellow and green luminescence components and the experimental defect emission for the three samples in (**a**). The defect emission of for the 200 °C hydrogen plasma treated undoped sample is also included. The CL measurements were performed at the temperature of 80 K and the electron energy is 5 keV.

Figure 5(a) shows the CL spectra of the as-grown and annealed ZnO films (grown with P(O_2_) = 0 Pa) with the electron energy of 5 keV (corresponding to the analysis depth of ~ 140 nm). Near band edge emission at 3.31 eV and defect emissions are observed in the spectra of all the three samples. Compared to the as-grown sample, the NBE intensity increases by a factor of 3 and 4.5 respectively after annealing at 700 °C and 900 °C. The defect emissions were fitted by two Gaussian peaks, a yellow luminescence (YL) positioned at 2.0 eV and a green luminescence at 2.4 eV (shown in Fig. 5(b)). Heating the film at 700 °C reduced the intensity of the YL relative to the GL while annealing at 900 °C strongly quenching the YL, leaving only the GL.

**Figure 6:**
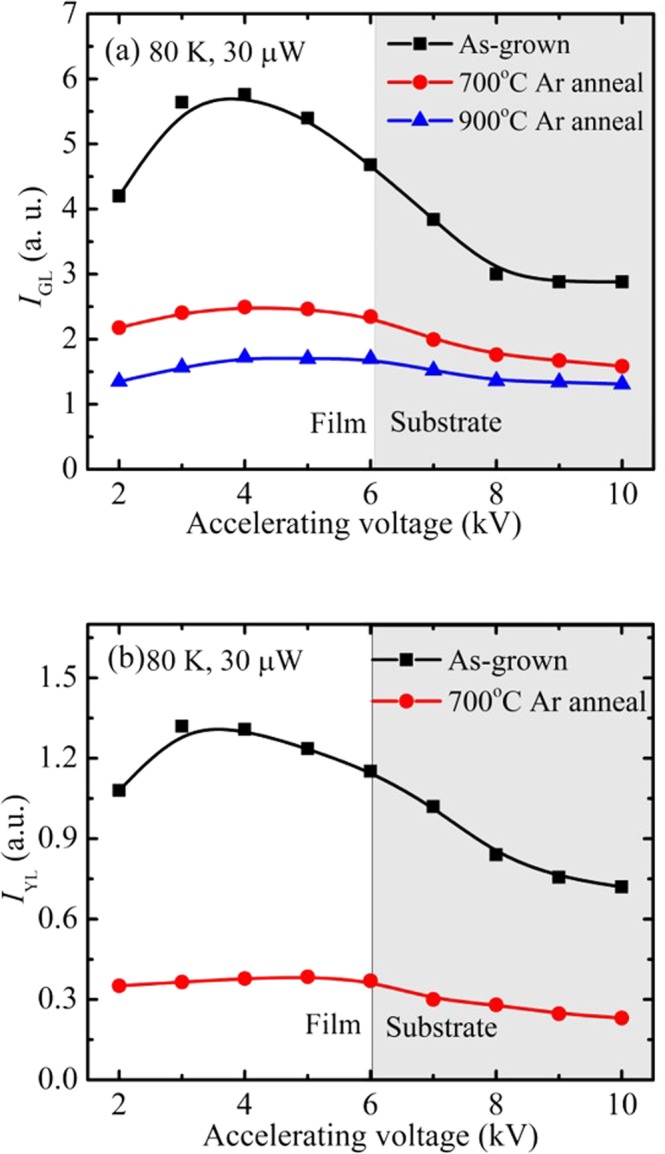
Intensities of the GL (**a**) and YL (**b**) for the defect emission as a function of the electron energy. The ZnO film and sapphire substrate regions relative to the CL sampling depth for various electron energies are also shown. $S={f}_{b}{S}_{b}+\sum _{i}\,{f}_{d,i}{S}_{d,i}$.

To study the in-depth distribution of GL and YL centres, depth-resolved CL spectra were acquired with a constant power (30 μW) by adjusting the beam current, *I*_B_, as the accelerating voltage was varied between 2 and 10 kV. Figure 6(a,b) show the I_GL_ and I_YL_ respectively of the fitted defect emission spectra as a function of accelerating voltage. The depth-resolved CL measurements were interpreted by modeling the in-depth electron energy loss using the Monte Carlo stimulation software CASINO^32^.

The measured and modeled depth-resolved CL results for the as-grown sample reveals the following: (i) the decrease of the GL and YL intensities near the ZnO surface at 2 kV for the as-grown sample suggests the presence of non-radiative recombination channels and/or a surface depletion layer due to surface band bending; and (ii) the GL and YL defect density decreases towards the sapphire interface in the as-grown sample. It is noticed that the GL intensity significantly drops after annealing at 900 °C. For the YL, its intensity significantly drops after 700 °C annealing and completely quenched at 900 °C, suggesting YL center is unstable at this temperature.

ZnO films were doped with H using a mild hydrogen plasma treatment at 200 °C. Following the incorporation of H, both the GL and YL are totally quenched (see Fig. 4(b)). As a H^+^ is a donor in ZnO, it would favor interacting with acceptor site rather than donor site^33^. In a previous CL study of O-rich and Zn-rich ZnO powder^33^, two distinct GL emissions with different peak positions at 2.30 and 2.53 eV were identified which were quenched and enhanced after the H-plasma treatment respectively. By considering the excitation-power dependencies, the GL at 2.30 eV in O-rich ZnO was assigned to band-to-V_Zn_ related acceptor and the GL at 2.53 eV in Zn-rich ZnO was ascribed to a band-to-V_O_ related donor. The YL in ZnO has been attributed to acceptors including both substitutional Li on Zn sites (Li_Zn_) and oxygen interstitials (O_*i*_)^33^. In the present study, H quenching of the YL precludes its assignment to Li-related centers as these centers are unaffected by hydrogen doping^34^ and consequently the YL observed in our films is assigned to O_i_-related centers.

The GL observed in this work is not associated with Cu impurities as it does not exhibit their signature fine structure at low temperature^34^. Moreover, as the GL was quenched following H-plasma treatment the involvement of V_O_ can also be ruled out, suggesting that the origin of the GL in our ZnO samples is related to acceptor-like V_Zn_-related defects^33^. Considering the energy states of intrinsic defects and their complexes in ZnO obtained from a density functional theory based pseudopential all-electron approach, GL has also been suggested to be originate from a V_Zn_V_O_ divacancy^35^. However, the *W-S* plots (Fig. 4) indicates that the GL observed in the present study cannot be produced by an isolated V_Zn_, a V_Zn_V_O_ divacancy or a 2V_Zn_V_O_ complex because the samples are dominated by the V_Zn_-nV_O_, n = 2,3 (Fig. 4), and the PAS signal is clearly distinguishable from those of V_Zn_, V_Zn_V_O_ and 2V_Zn_V_O_. A similar negative correlation between the GL and isolated V_Zn_ has also been reported by Chen *et al*.^18^, who studied the electron irradiated ZnO single crystal by PAS and CL. This work argued that V_Zn_ would instead act as non-radiative recombination centers. Using hybrid density functional theory calculations, Frodason *et al*.^36^ reported that an isolated V_Zn_ was a deep polaronic acceptor. Furthermore using a one-dimensional configuration coordinate model to simulate the luminescence peak and lineshape for an isolated V_Zn_, it was concluded that this defect was unlikely to be the cause of the GL.

In the present study, we cannot provide an unequivocal assignment for the origin of the observed GL intensity, which is quenched by H-plasma treatment and anneals out at 900 °C (Fig. 6(a)). The latter finding is indeed correlated with the experimental PAS results showing the formation of the vacancy cluster containing more V_Zn_ as relative to V_Zn_-nV_O_ (n = 2, 3) which occurs at the same annealing temperature. These results open the door for further discussion. If the V_Zn_-nV_O_ is considered to be the cause of the GL, the H-plasma treatment may incorporate the H into the V_Zn_ site of the V_Zn_-nV_O_ cluster. The new H decorated vacancy cluster may become optically inactive and thus quenches the GL. It is also important to note that with the reduction in the GL intensity following the formation of the new vacancy cluster which is relatively V_Zn_ rich after annealing at 900 °C, no new defect emission is observed and the NBE emission is enhanced. This is consistent with the newly formed vacancy cluster not being optically active and no longer competing with the NBE radiative recombination pathways. Nevertheless, the origin of the GL observed in the present study cannot be assigned at this time to a specific defect center and requires further investigation.

The formation energies of monovacancies V_Zn_ are lower than those of the vacancy clusters 2V_Zn_-V_O_ and V_Zn_-2V_O_ by 2.5–3.1 eV^37^. However, in this work, the vacancy clusters become the dominant defect rather than the V_Zn_. The dissociation energy of a vacancy cluster is equal to the sum of its binding energy and the diffusion barrier of the corresponding monovacancy. For n-type ZnO, the dissociation energies for the V_Zn_V_O_, 2V_Zn_-V_O_ and V_Zn_-2V_O_ are high^37^. This implies that once the vacancy cluster is aggregated via the diffusion of the monovacancies, they are energetically difficult to dissociate at the relatively low temperatures^37^. This means that the formation of the vacancy cluster is driven by the kinetic effect rather than by thermodynamic equilibrium. Thermodynamic process of defects governed by the formation energy of the defects is dominant at relatively higher temperatures typically above 900 °C. In the present study, the substrate temperature is 600 °C during the PLD growth. At this relatively low growing temperature, kinetic processes on the growth surface would become dominant, which would lead to the dominance of vacancy cluster rather than monovacancy as the dissociation energy of vacancy cluster is high. The annealing of the samples at 900 °C is also not a thermodynamic equilibrium process as there is excessive V_Zn_ creation at the film/substrate boundary due to Zn out-diffusion. The excessive V_Zn_ creation during annealing may be the reason why instead of simply annealing out, vacancy cluster containing more V_Zn_ as relative to V1 is formed. However the exact dynamic and interaction of different kinds of vacancy type defects is not known in the present study and require further investigation.

Secondary ion mass spectroscopy (SIMS) study was conducted to obtain the hydrogen concentration of the as-grown and 900 °C annealed undoped ZnO samples. They are found to be respectively 7 × 10^18^ cm^−3^ and 3 × 10^18^ cm^−3^, which are close to the electron concentrations of 8 × 10^18^ cm^−3^ and 4 × 10^18^ cm^−3^ for the as-grown and 900 °C annealed samples. The n-type conductivity of the undoped ZnO could be due to the hydrogen impurity as it is known to be a shallow donor^38^. It is also noticed that the electron concentration for the undoped sample grown with P(O_2_) = 0 Pa increase from ∼2 × 10^18^ cm^−3^ to ∼4 ×10^18^ cm^−3^ while the annealing temperature increases from 750 °C to 900 °C (see Fig. 2). For the sample grown with P(O_2_) = 1 Pa, similar electron concentration and mobility increase are also observed as the annealing temperature increases from 750 °C to 900 °C. It is also observed that the electron mobilities also increases upon the 900 °C annealing. The increase of electron concentration and mobility correlate with the observation in the W-S plot that the formation of the vacancy cluster with more V_Zn_ as relative to V1 which occurs at the same annealing temperature. This could be explained if the V1 cluster is initially a compensating defect and the new defect formed after annealing at 900 °C is not electrical active.

## Conclusion

Zn-vacancy related defects in ZnO films grown by PLD method were systemically studied using the PAS and CL. PAS study shows that vacancy cluster V1 (which is possibly V_Zn_-nV_O_ with n = 2, 3) is the dominant V_Zn_-related defect in the as-grown undoped ZnO samples grown with relatively low P(O_2_). Annealing at 900 °C changes the microstructure of the vacancy cluster becoming containing more V_zn_, whereas excessive V_Zn_ are created by Zn out-diffusion at the film/substrate interface induced by annealing. Growing the undoped ZnO samples at higher P(O_2_) would favor the formation of vacancy cluster with more V_Zn_ comparing to V1. The domination of vacancy cluster rather than the isolated V_Zn_ with lower formation energy could be due to the vacancy aggregation resulted from the vacancy diffusion and the high dissociation energies of the vacancy cluster. GL and YL were observed in the CL study of the samples and they anneal out respectively at 750 °C and 900 °C and both emissions were found to be quenched by H-plasma treatment. The GL found in this study cannot be assigned to isolated V_Zn_, V_O_ or the V_Zn_V_O_ centers.

## Supplementary information

On-line supplemental information

## References

[1] Özgür Ü (2005). A comprehensive review of ZnO materials and devices. J. Appl. Phys..

[2] McCluskey MD, Jokela S (2009). Defects in ZnO. J. Appl. Phys..

[3] Look DC, Claflin B (2004). P‐type doping and devices based on ZnO. Phys. Stat. Sol. (b).

[4] Liu L (2012). p-Type conductivity in n-doped ZnO: the role of the N_Zn_– V_O_ complex. Phys. Rev. Lett..

[5] Reynolds J (2013). Shallow acceptor complexes in p-type ZnO. Appl. Phys. Lett..

[6] Tuomisto F, Ranki V, Saarinen K, Look DC (2003). Evidence of the Zn vacancy acting as the dominant acceptor in n-type ZnO. Phys. Rev. Lett..

[7] Look DC (2011). Self-compensation in semiconductors: The Zn vacancy in Ga-doped ZnO. Phys. Rev. B.

[8] Limpijumnong S, Zhang S, Wei S-H, Park C (2004). Doping by large-size-mismatched impurities: the microscopic origin of arsenic-or antimony-doped p-type zinc oxide. Phys. Rev. Lett..

[9] Luo C (2018). Sb-related defects in Sb-doped ZnO thin film grown by pulsed laser deposition. J. Appl. Phys..

[10] Khalid M (2009). Defect-induced magnetic order in pure ZnO films. Phys. Rev. B.

[11] Herng T (2010). Room-temperature ferromagnetism of Cu-doped ZnO films probed by soft X-ray magnetic circular dichroism. Phys. Rev. Lett..

[12] Brauer G (2006). Defects in virgin and N+-implanted ZnO single crystals studied by positron annihilation, Hall effect, and deep-level transient spectroscopy. Phys. Rev. B.

[13] Dong Y, Tuomisto F, Svensson BG, Kuznetsov AY, Brillson LJ (2010). Vacancy defect and defect cluster energetics in ion-implanted ZnO. Phys. Rev. B.

[14] Zubiaga A (2008). Mechanisms of electrical isolation in O+-irradiated ZnO. Phys. Rev. B.

[15] Tuomisto F (2013). Nitrogen and vacancy clusters in ZnO. J. Mater. Res..

[16] Krause-Rehberg, R. & Leipner, H. S. *Positron annihilation in semiconductors: defect studies*. Vol. 127 (Springer Science & Business Media, 1999).

[17] Tuomisto F, Makkonen I (2013). Defect identification in semiconductors with positron annihilation: experiment and theory. Rev. Mod. Phys..

[18] Chen Z (2007). Thermal evolution of defects in as-grown and electron-irradiated ZnO studied by positron annihilation. Phys. Rev. B.

[19] Brauer G (2009). Identification of Zn-vacancy–hydrogen complexes in ZnO single crystals: A challenge to positron annihilation spectroscopy. Phys. Rev. B.

[20] Brunner, S., Puff, W., Balogh, A. G. & Mascher, P. In *Mater. Sci. Forum*. 141–143 (Trans Tech Publications Ltd., Zurich-Uetikon, Switzerland).

[21] Selim F, Weber M, Solodovnikov D, Lynn K (2007). Nature of native defects in ZnO. Phys. Rev. Lett..

[22] Johansen K (2011). H passivation of Li on Zn-site in ZnO: Positron annihilation spectroscopy and secondary ion mass spectrometry. Phys. Rev. B.

[23] Wang Z, Su S, Ling FC-C, Anwand W, Wagner A (2014). Thermal evolution of defects in undoped zinc oxide grown by pulsed laser deposition. J. Appl. Phys..

[24] Venkatachalapathy V, Galeckas A, Zubiaga A, Tuomisto F, Kuznetsov AY (2010). Changing vacancy balance in ZnO by tuning synthesis between zinc/oxygen lean conditions. J. Appl. Phys..

[25] Zubiaga A (2005). Zinc vacancies in the heteroepitaxy of ZnO on sapphire: Influence of the substrate orientation and layer thickness. Appl. Phys. Lett..

[26] Särkijärvi S (2014). Effect of growth temperature on the epitaxial growth of ZnO on GaN by ALD. J. Crystal Growth.

[27] Makkonen I, Korhonen E, Prozheeva V, Tuomisto F (2016). Identification of vacancy defect complexes in transparent semiconducting oxides ZnO, In2O3 and SnO2. J. Phys.: Condens. Mat..

[28] Van Veen A (1995). VEPFIT applied to depth profiling problems. Appl. Surf. Sci..

[29] Saarinen K, Suski T, Grzegory I, Look DC (2001). Thermal stability of isolated and complexed Ga vacancies in GaN bulk crystals. Phys. Rev. B.

[30] Wang R, Gu Q, Ling C, Ong H (2008). Studies of oxide/ZnO near-interfacial defects by photoluminescence and deep level transient spectroscopy. Appl. Phys. Lett..

[31] Stehr JE (2014). Zinc-vacancy–donor complex: a crucial compensating acceptor in ZnO. Phys. Rev. Appl..

[32] Drouin D (2007). CASINO V2. 42—a fast and easy‐to‐use modeling tool for scanning electron microscopy and microanalysis users. Scanning.

[33] Ton-That C, Weston L, Phillips M (2012). Characteristics of point defects in the green luminescence from Zn-and O-rich ZnO. Phys. Rev. B.

[34] Dingle R (1969). Luminescent transitions associated with divalent copper impurities and the green emission from semiconducting zinc oxide. Phys. Rev. Lett..

[35] Vidya R (2011). Energetics of intrinsic defects and their complexes in ZnO investigated by density functional calculations. Phys. Rev. B.

[36] Frodason Y, Johansen K, Bjørheim T, Svensson B, Alkauskas A (2017). Zn vacancy as a polaronic hole trap in ZnO. Phys. Rev. B.

[37] Bang J, Kim Y-S, Park C, Gao F, Zhang S (2014). Understanding the presence of vacancy clusters in ZnO from a kinetic perspective. Appl. Phys. Lett..

[38] Van de Walle CG (2000). Hydrogen as a cause of doping in zinc oxide. Phys. Rev. Lett..

