# Supplementary material for: Vacancy cluster in ZnO films grown by pulsed laser deposition
Source: Sci Rep. 2019 Mar 5;9:3534. doi: 10.1038/s41598-019-40029-3 (PMC6401145; doi:10.1038/s41598-019-40029-3)
Supplement: Supplementary file 1 — On-line supplemental information [file 41598_2019_40029_MOESM1_ESM.docx]

**Vacancy cluster in ZnO films grown by pulsed laser deposition**

Zilan Wang^1^, Caiqin Luo^1^, W. Anwand^2^, A. Wagner^2^, M. Butterling^2^, M. Azizar Rahman^3^, Matthew R. Phillips^3^, Cuong Ton-That^3^, M. Younas^1,4^, Shichen Su^1,5^, Francis Chi-Chung Ling^1,^*

*^1^ Department of Physics, The University of Hong Kong, Pokfulam Road, P. R. China*

*^2^ Institute of Radiation Physics, Helmholtz-Zentrum Dresden-Rossendorf, Bautzner Landstr. 400, 01328 Dresden, Germany*

*^3^ School of Mathematical and Physical Sciences, University of Technology Sydney, Ultimo, NSW 2007, Australia*

*^4^ EMMG, Physics Division, PINSTECH, P.O. Nilore, Islamabad, Pakistan*

*^5^  Institute of Optoelectronic Material and Technology, South China Normal University, Guangzhou 510631, P. R. China*

^*^ Contact author: [ccling@hku.hk](mailto:ccling@hku.hk)

**On-line Supplemental Information**

**S1. XRD Study**


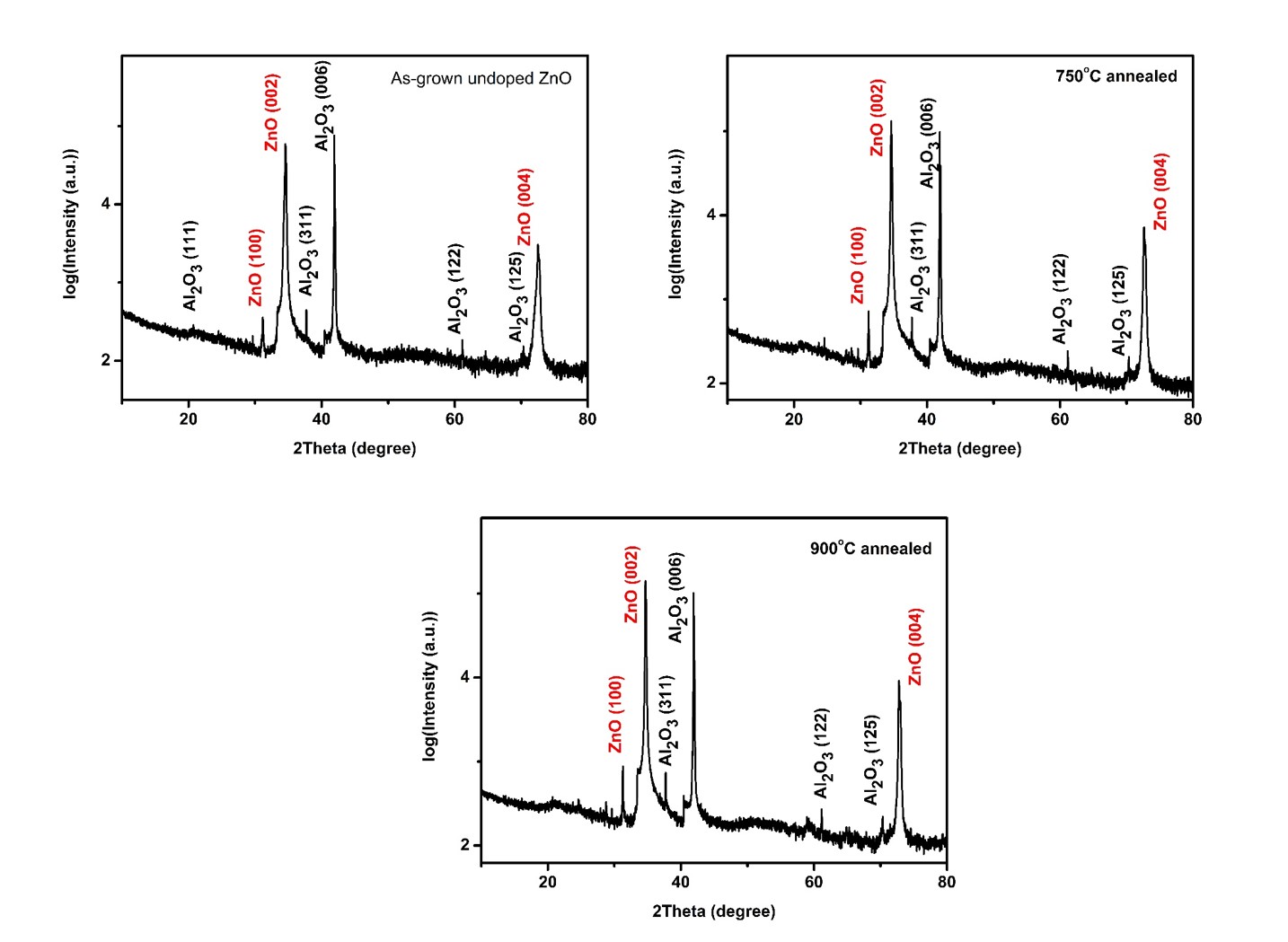


Figure S1 The XRD spectra of the as-grown, 750 ^o^C and 900 ^o^C annealed undoped ZnO sample grown with the oxygen pressure of P(O_2_)=0.02 Pa.

The XRD spectra of the as-grown, 750 ^o^C annealed and 900 ^o^C annealed undoped ZnO sample grown with the oxygen pressure of 0.02 Pa is shown in Figure S1. Two major ZnO related peaks namely the ZnO (002) at ~34.5^o^, ZnO (004) ) at ~72.1^o^ are found. Another ZnO related peak (100) is also observed, though with a negligible intensity as compared to that of the dominant (002) peak (~150 times smaller in intensity). This shows that the ZnO films have the single-phase wurtzite structure with the c-axis as the preferential orientation. Annealing the samples at 750 ^o^C and 900 ^o^C do not introduce new phase. Similar features were also found in the XRD spectra of all the other ZnO films grown with the different oxygen pressures.
